# p53 Dependent Centrosome Clustering Prevents Multipolar Mitosis in Tetraploid Cells

**DOI:** 10.1371/journal.pone.0027304

**Published:** 2011-11-04

**Authors:** Qiyi Yi, Xiaoyu Zhao, Yun Huang, Tieliang Ma, Yingyin Zhang, Heli Hou, Howard J. Cooke, Da-Qing Yang, Mian Wu, Qinghua Shi

**Affiliations:** 1 Hefei National Laboratory for Physical Sciences at Microscale and School of Life Sciences, University of Science and Technology of China, Hefei, Anhui, China; 2 MRC Human Genetics Unit and Institute of Genetics and Molecular Medicine, Western General Hospital, Edinburgh, United Kingdom; 3 Sanford Research/University of South Dakota, Sanford Health, Sioux Falls, South Dakota, United States of America; Roswell Park Cancer Institute, United States of America

## Abstract

**Background:**

p53 abnormality and aneuploidy often coexist in human tumors, and tetraploidy is considered as an intermediate between normal diploidy and aneuploidy. The purpose of this study was to investigate whether and how p53 influences the transformation from tetraploidy to aneuploidy.

**Principal Findings:**

Live cell imaging was performed to determine the fates and mitotic behaviors of several human and mouse tetraploid cells with different p53 status, and centrosome and spindle immunostaining was used to investigate centrosome behaviors. We found that p53 dominant-negative mutation, point mutation, or knockout led to a 2∼ 33-fold increase of multipolar mitosis in N/TERT1, 3T3 and mouse embryonic fibroblasts (MEFs), while mitotic entry and cell death were not significantly affected. In p53^-/-^ tetraploid MEFs, the ability of centrosome clustering was compromised, while centrosome inactivation was not affected. Suppression of RhoA/ROCK activity by specific inhibitors in p53^-/-^ tetraploid MEFs enhanced centrosome clustering, decreased multipolar mitosis from 38% to 20% and 16% for RhoA and ROCK, respectively, while expression of constitutively active RhoA in p53^+/+^ tetraploid 3T3 cells increased the frequency of multipolar mitosis from 15% to 35%.

**Conclusions:**

p53 could not prevent tetraploid cells entering mitosis or induce tetraploid cell death. However, p53 abnormality impaired centrosome clustering and lead to multipolar mitosis in tetraploid cells by modulating the RhoA/ROCK signaling pathway.

## Introduction

Aneuploidy, the condition in which a cell has extra or missing chromosomes, is the most common characteristic of human cancers and is linked to the progressive development of high-grade, invasive tumors. Despite this crucial role, its origins remain elusive. A long-standing hypothesis is that a genetically metastable tetraploid intermediate could facilitate the development of aneuploid malignancies [1], [2], [3]. This assumption is supported by researches in both cultured cells [4] and animal models [5]. Furthermore, some studies demonstrate that tetraploidy arises as an early step in tumorigenesis where its presence is associated with the inactivation of the tumor suppressor p53. This precedes the formation of aneuploid cells [6], [7], [8]. Further support comes from the observation that p53^-/-^ tetraploid mouse mammary epithelial cells (MMECs) induced malignant mammary epithelial cancers after subcutaneous injection into nude mice, while p53^-/-^ diploid and p53^+/+^ tetraploid MMECs did not [9].

p53 may regulate the level of tetraploidy and/or its deleterious effects through multiple mechanisms. First, it has been shown that loss of p53 facilitates spontaneous generation of tetraploid cells in various mouse and human cell lines in culture [10], [11]. Second, p53 could influence the fate of tetraploid cells once formed. It is reported that p53 induced apoptosis of tetraploid cells in cultured cancer cell lines and primary mouse mammary epithelial cells [12], [13]. It has been suggested that there might be a p53 related tetraploidy checkpoint that could permanently block the proliferation of tetraploid cells [4], [14], [15]. However, this assumption has been increasingly contested by other findings that show this inhibition could be a side effect of drugs used to induce tetraploidy [16], [17]. Third, p53 may influence the survivability of cells with extra centrosomes, such as tetraploid cells and part of their daughter cells. One research showed that the descendant cells from p53^-/-^ HCT116 tetraploidy have a higher proportion of cells with extra centrosomes than that from p53^+/+^ HCT116 tetraploidy after a long time culturing, which induced more multipolar mitosis in p53^-/-^ cells [18]. Another study showed that p53-driven apoptosis occurs as a checkpoint mechanism to prevent accumulation of cells with extra centrosomes [19].

It has long been proposed that supernumerary centrosomes could lead to multipolar mitosis and aneuploidy and thereby contribute to clonal evolution. This notion has been recently validated experimentally [20]. However, cells with supernumerary centrosomes, such as tetraploid cells, could maintain bipolar spindles by two strategies: 1) Inactivation of extra centrosomes making them unable to organize microtubules; 2) clustering of extra centrosomes into two microtubule organizing centers [21]. To identify comprehensively genes required for suppressing multipolar mitoses, genome-wide RNAi screens were performed in Drosophila S2 cells and oral squamous cell carcinoma cell line SCC114 [22], [23]. Three groups of proteins were discovered by these studies. The first group proteins contains members of the spindle assembly checkpoint (SAC) required for ensuring adequate time for converting to a bipolar spindle prior to anaphase. The second group of proteins contains microtubule-binding proteins with roles in spindle pole focusing. The third group contains proteins involved in the organization and regulation of the actin cytoskeleton, such as the formin Form3/INF2 and GTPases. The Rho family of GTPases, key regulators of actin cytoskeleton reorganization, has been implicated in different stages of tumour progression. For example, constitutively active Rho GTPases can cause cell transformation [24]. Further investigations have shown that activated Rho GTPases induce tumour development in experimental mouse models [25], and activation of RhoA contributes to distant lung metastasis in vivo [26]. However, genes encoding Rho GTPases have rarely been found mutated in human cancers, where it seems that their activities are deregulated [27], [28], [29], [30]. This suggests that alterations of other genes might account for the functional modifications of Rho GTPases which accompany actin cytoskeleton remodeling during the neoplastic process. As an example, mutant p53 or loss of p53 induces overactivation of RhoA [31], [32], [33], [34], [35]. In this study, we have demonstrated that overactivated RhoA accounted for increased multipolar mitosis in p53 abnormal tetraploid cells, through impairing the ability of centrosome clustering. This provides an alternative explanation for arising of aneuploidy during tumor initiation and the frequently observed coexistence of p53 abnormalities, genomic instability and aneuploidy in human tumors.

## Results

### p53 abnormality has limited effect on the fates of tetraploid cells

**Figure 1:**
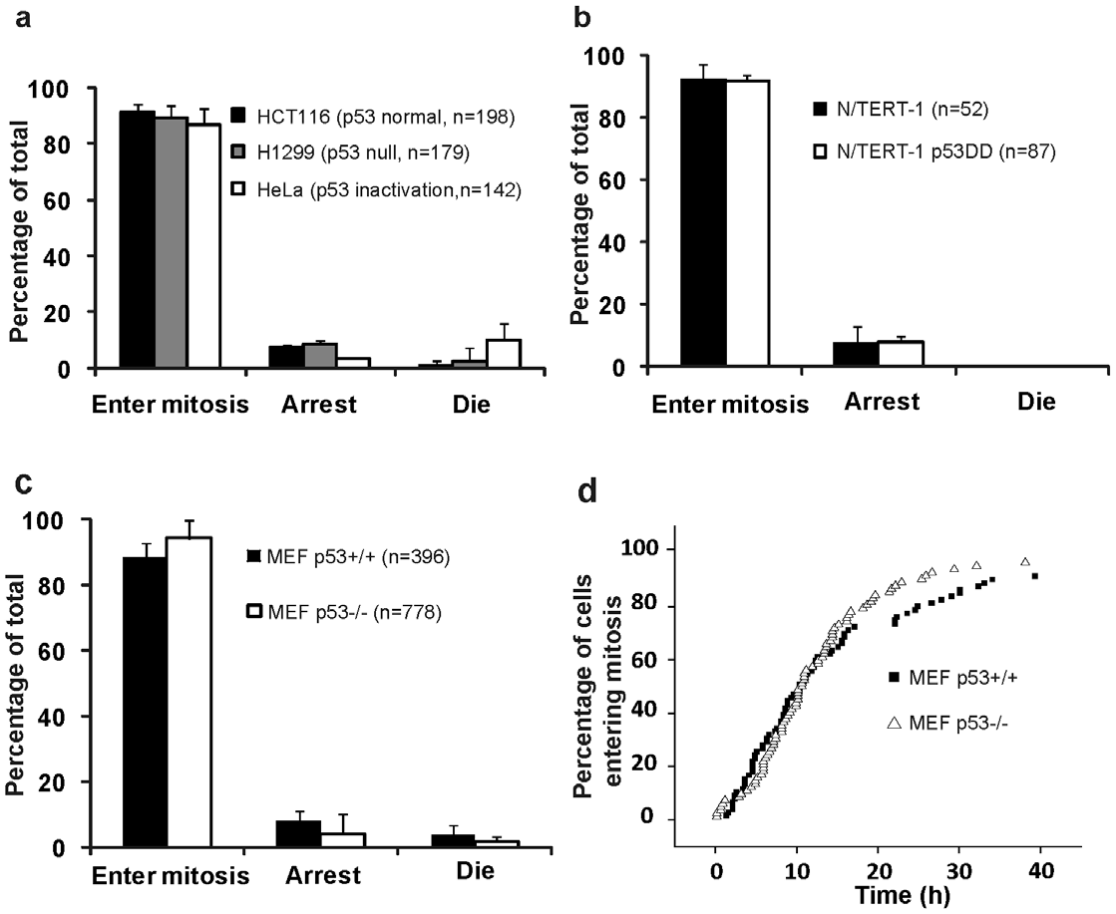
Effect of p53 on the fates of tetraploid cells. Cells were treated with Cyto-B to obtain tetraploid cells. After washing, cells were cultured in drug-free culture medium, and then tracked by live cell imaging. (A, B, C) Fates of tetraploid cells were detected by live cell imaging of cells with different p53 status in 3 (A), 2 (B), 5 (C) independent experiments. n, the number of cells analyzed. **(**D) p53 could not delay mitosis of tetraploid MEFs. Representative time course of entering mitosis of p53^+/+^ and p53^-/-^ tetraploid cells from one experiment are shown.

We examined the effects of p53 on the fates of tetraploid cells by long-term live cell imaging of cells with different p53 status. We first tracked cell proliferation in a human colon cancer cell line HCT116 (wild type p53), a human non-small cell lung carcinoma cell line H1299 (p53 null) and a human cervical cancer cell line HeLa (p53 inactivated by E6) (from the IARC TP53 Database, URL: http://www-p53.iarc.fr). To better visualize the cell nuclei, all cell lines were transfected with a eukaryotic expression plasmid encoding a mCherry fluorescent protein- H2B fusion protein. After the induction of binucleated cells with cytochalasin B (Cyto-B), live cell imaging on these cells was conducted. As shown in Fig. 1A, 91±3% of binucleated HCT116 cells, 89±4% of binucleated H1299 cells and 86±6% of binucleated HeLa cells entered mitosis. Therefore, most binucleated cancer cells could enter mitosis, irrespective of the status of p53. These cancer cells are of different origins and have different genetic backgrounds, and they may have defective signaling pathways, so the results from experiments using these cells may not be comparable. Thus, we used N/TERT-1, a primary diploid human keratinocytic cell line immortalized with telomerase [36], [37], and the p53 defective counterpart N/TERT-1 p53DD over-expressing dominant negative p53 [36]. After treatment with Cyto-B for 24 h, their mitotic capacity was investigated by live cell imaging. We observed that 92±5% of N/TERT-1 binucleated (tetraploid) cells and 92±2% of N/TERT-1 p53DD binucleated (tetraploid) cells entered mitosis by the end of the movie. The remaining cells arrested in interphase and cell death was not observed (Fig. 1B). To avoid any further concerns about the peculiarities of immortalized cell lines during the long term in vitro culture, we turned to primary mouse embryonic fibroblasts (MEFs) and their p53-deficient counterparts (p53^-/-^ MEFs). With 0.5 µg/ml Cyto-B treatment for 12 h, 30∼40% of MEFs were binucleated (tetraploidy). We observed that 88±4% of p53^+/+^ and 94±5% of p53^-/-^ MEF tetraploid cells entered mitosis (Fig. 1C). The proportion of arrested cells in p53^+/+^ and p53^-/-^ MEF tetraploid cells were 8±3% and 4±6%, respectively. Only 4±3% of p53^+/+^and 2±1% of p53^-/-^ MEF tetraploid cells died, and it was not significantly different (p>0.05, 2×2 *χ^2^* test). To investigate the effects of p53 on the cell cycle in the tetraploid population, we plotted the proportions of tetraploid cells entering mitosis against time. The p53^+/+^ MEF tetraploid cells were found to have the same dynamics as p53^-/-^ MEF tetraploid cells in entering mitosis (Fig. 1D).

These results provide a functional demonstration that p53 did not significantly influence the fate of tetraploid cells especially has no effect on cell death.

### p53 abnormality promotes multipolar mitosis of tetraploid cells

**Figure 2:**
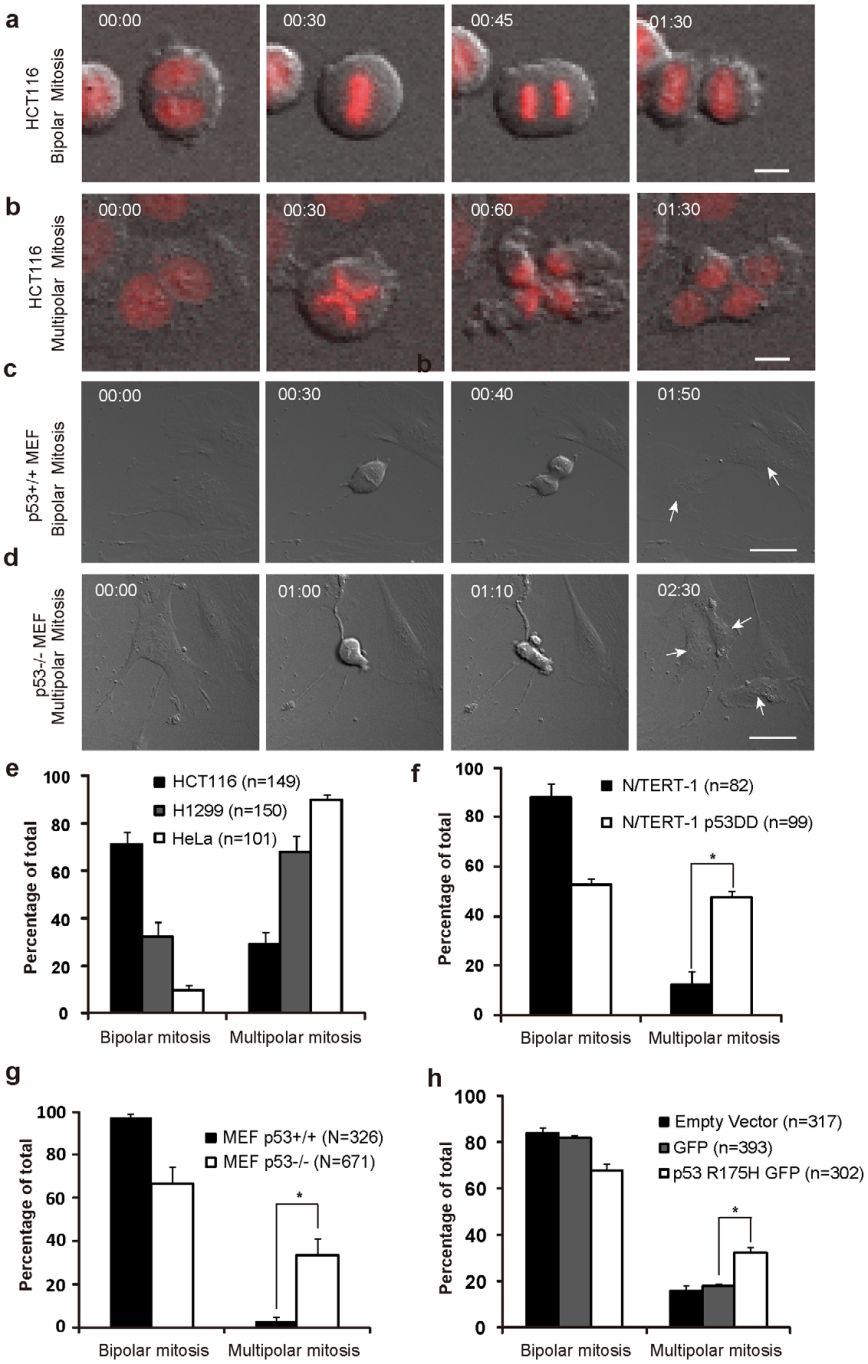
Loss function of p53 promotes multipolar mitosis in tetraploid cells. (A-D) Selected serial images from time-lapse records showing examples of: (A) Bipolar mitosis of a tetraploid HCT116 cell; (B) Multipolar mitosis of a tetraploid HCT116 cell; Chromosomal material is shown in Red. Bar = 10 µm (A,B); (C) Bipolar mitosis of a tetraploid p53^+/+^ MEF cell; (D) Multipolar mitosis of a tetraploid p53^-/-^ MEF cell. Bar = 50 µm (C,D). Time counted from cell entering mitosis is presented as h:min at top-left. Arrows point to daughter cells of bipolar mitosis (C) and tripolar mitosis (D). (E-G) Comparisons of the percentage of the tetraploid cells with the indicated mitotic polarity between p53 normal and abnormal cells, respectively. (H) The frequency of multipolar mitosis in tetraploid cells increased after p53 R175H expression in 3T3 cells. Mitotic polarity of cells was examined by live cell imaging after transient transfection of indicated plasmids followed by 1.5 µg/ml Cyto-B treatment for 12 h. * P<0.001, 2×2 χ^2^ test. Error bars are standard deviation, from results of 3 (E), 2 (F), 5 (G), 3 (H) independent experiments. n, the number of cells analyzed.

As most tetraploid cells (>86%) could enter mitosis even in p53 normal cells, we next investigated the mitotic polarity of p53 normal and abnormal tetraploid cells which is strongly correlated to the genesis of aneuploidy (Fig. 2A,B,C,D). As shown in Fig. 2E, multipolar mitosis occurred much more frequently in binucleated cells with defective p53 (90±2% for HeLa and 68±6% for H1299) than in p53 normal binucleated cells (29±5% for HCT116). In N/TERT-1 tetraploid cells, disruption of normal p53 function by p53 mutant resulted in a significant increase in the incidence of multipolar mitosis (Fig. 2F, 47±3% vs. 12±5%, p<0.05, 2×2 *χ^2^* test). p53^-/-^ MEF tetraploid cells also showed a significant increase in the incidence of multipolar mitosis (33±8%) compared to the p53^+/+^ counterparts (1±2%) (Fig. 2G, p<0.001, 2×2 *χ^2^* test). Amino acid residue 175 is one of the most frequently mutated sites of the p53 protein. We then asked whether p53 R175H could also promote multipolar mitosis in tetraploid cells. Given that primary MEFs are hard to be transfected, we turned to NIH 3T3 cells, a murine fibroblast cell line with normal p53 protein (Fig. S1) [38]. We found that induction of a p53 R175H-GFP fusion protein in 3T3 cells significantly increased the proportion of multipolar mitosis (32±3% vs. 18±1% for cells expressing GFP only (p<0.001, 2×2 *χ^2^* test, Fig. 2H)) in tetraploid cells. Thus, these observations indicate that abnormalities of p53 promote multipolar mitosis in tetraploid cells.

### p53 deficient tetraploid cells are defective in clustering extra centrosomes

**Figure 3:**
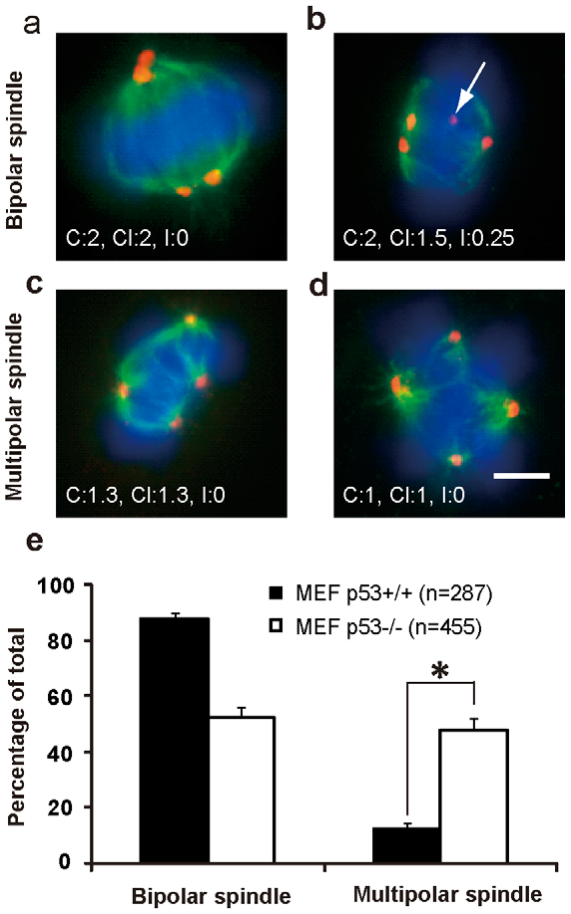
The absence of p53 promotes the formation of multipolar spindles in tetraploid cells. p53^+/+^ and p53^-/-^ MEF cells cultured on glass coverslips coated with 0.1% gelatin were treated with 0.5 µg/ml Cyto-B for 12 h. After washing, cells were cultured for additional 8 h in drug-free culture medium and subjected to immunofluorescence detection of mitotic spindles (α-tubulin staining, green fluorescence) and centrosomes (γ-tubulin staining, red fluorescence). Nuclei were counterstained with DAPI (emitting in blue). Representative images of each spindle type and C, Cl and I values are shown respectively on down-left. (A) A bipolar spindle with two centrosomes clustered loosely on lower pole or closely on upper pole. (B) A bipolar spindle showing two centrosomes clustered on one pole and one centrosome inactivated (white arrow). (C) A tripolar spindle with two centrosomes loosely clustered on one pole. (D) A tetrapolar spindle with one centrosome on every pole. Bar = 10 µm (A-D). (E) Analysis of spindle polarity at metaphase of cells with extra centrosomes (* P<0.001, 2×2 χ^2^ test). Error bars are standard deviation, from results of 3 independent experiments. n, the number of cells analyzed.

Theoretically, centrosome clustering and centrosome inactivation could be alternative mechanisms allowing cells with extra centrosomes, such as tetraploid cells, to maintain bipolar mitosis [21], [39]. To investigate multipolar mitosis in p53 abnormal tetraploid cells, we studied centrosome behaviors at metaphase in tetraploid cells. We identified centrosomes by immunostaining γ-tubulin and mitotic spindles using α-tubulin. We used three parameters, C value (the number of centrosomes/the number of spindle poles), Cl value (the number of activated centrosomes/the number of spindle poles) and I value (the number of inactivated centrosomes/total number of centrosomes), to indicate the ability of maintaining bipolar spindle, clustering extra centrosomes and inactivating extra centrosomes of a cell, respectively. We observed that 36±2% HCT116 tetraploid cells displayed multipolar spindles, in HeLa tetraploid cells the percentage was 93±2%. In p53^-/-^ tetraploid MEFs, 48±4% were multipolar, compared to 12±2% in p53^+/+^ tetraploid MEFs (Fig. 3E, p<0.001, 2×2 *χ^2^* test), consistent with previous results from live cell imaging. We found that both centrosome clustering and centrosome inactivation existed to sustain bipolar spindle in tetraploid MEFs (Fig. 3A,B,C,D). 93.7±1.6% of p53^+/+^ tetraploid MEFs displayed centrosome clustering while only 73.0±5.2% of p53^-/-^ counterparts did the same so. Moreover, p53^-/-^ tetraploid MEFs had much a higher proportion of cells without clustered or inactivated centrosomes than p53^+/+^ tetraploid MEFs (Table 1). However, the proportion of cells with centrosome inactivation was similar, 26.1±8.5% in p53^+/+^ tetraploid MEFs and 28.8±2.4% in p53^-/-^ counterparts. C value and Cl value in p53^+/+^ MEFs were significantly higher than those in p53^-/-^ MEFs (C value 2.06±0.60 vs. 1.74±0.59; Cl value 1.90±0.58 vs. 1.60±0.59; p<0.001, t-test, Table 2), indicating that the ability to cluster extra centrosomes was impaired when p53 was lost. The I value was similar in MEF cells with different p53 genotypes, indicating that p53 was not involved in centrosome inactivation. To investigate more precisely the centrosome behavior in tetraploid cells of different p53 genotype, we only compared tetraploid cells with four centrosomes at metaphase. These p53^-/-^ tetraploid MEFs displayed much more multipolar spindles than p53^+/+^ tetraploid MEFs (data not shown), losing the ability to cluster rather than inactivate extra centrosomes (Table S1, S2).

**Table 1:** Centrosome clustering was impaired in p53^-/-^ MEF cells with extra centrosomes.

	No. of cells analyzed	% (Mean ± s.d.)			
		Centrosome inactivation	Centrosome clustering	Both	Neither
p53^+/+^ MEF	287	26.1±8.5	91.3±5.0	21.2±5.8	3.8±1.7
p53^-/-^ MEF	455	28.8±2.4	73.0±5.2*	15.4+2.0	13.6±5.6 *

Cells were treated with Cyto-B (0.5 µg/ml) for 12 h, then released for 8 h, and stained with antibodies against γ-tubulin and α-tubulin for centrosomes and microtubules, respectively. Nuclei were stained with DAPI. Note that only cells with more than 3 centrosomes at metaphase were analyzed. The percentage of cells with indicated mechanism was shown separately. Mean ± s.d. from three independent experiments.

*p<0.001, 2×2 χ^2^ test, compared with MEF p53^+/+^ cells.

**Table 2:** Centrosome behavior of MEF cells with extra centrosomes.

	No. of cells analyzed	Mean±s.d.		
		C value a	Cl value b	I value c
p53^+/+^ MEF	287	2.06±0.60	1.90±0.58	0.07±0.13
p53^-/-^ MEF	455	1.74±0.59 *	1.60±0.59 *	0.08±0.14

*p<0.001, t-test, compared with MEF p53^+/+^ cells.

a C value ( the number of centrosomes/the number of spindle poles) indicates the ability of maintaining bipolar spindle in cells with extra centrosomes;

b Cl value (the number of activated centrosomes/the number of spindle poles) indicates the ability of clustering centrosomes in cells with extra centrosomes;

c I value (the number of inactivated centrosomes/the number of total centrosomes) indicates the ability of inactivating extra centrosomes in cells with extra centrosomes.

Collectively, these results indicate that loss of p53 function decreases the frequency of centrosome clustering and promotes multipolar mitosis in tetraploid cells.

### Over-activated RhoA contributes to multipolar mitosis in p53 deficient tetraploid cells

**Figure 4:**
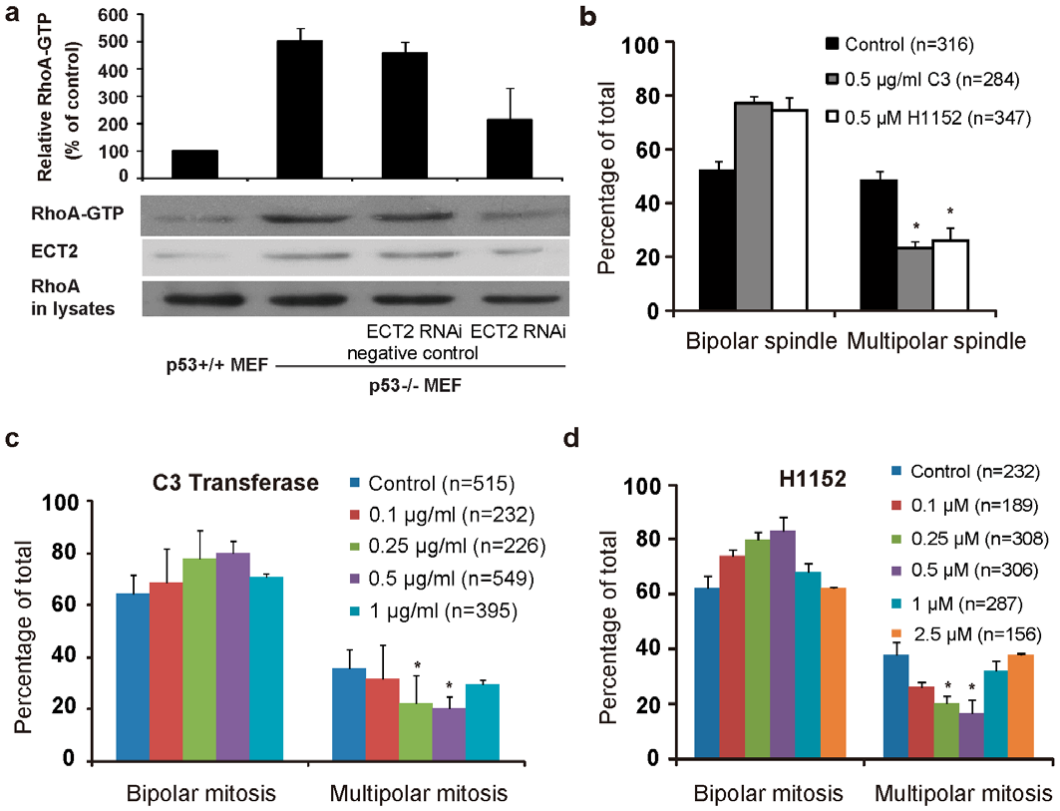
Over-activated RhoA contributes to multipolar spindle formation in p53^-/-^ tetraploid cells. (A) The endogenous RhoA activities (RhoA-GTP) in the p53^+/+^ and p53^-/-^ MEF cells were assayed after indicated treatment. The lysates were subject to GST-Rhotekin pull-down analysis. The amount of RhoA-GTP was detected by Western blotting of the glutathione-agarose coprecipitated with anti-RhoA antibody and was normalized to that of RhoA in wild-type MEFs. The results are shown as the mean ± s.d. of three experiments. (B) The frequency of multipolar spindle at metaphase in MEF cells with extra centrosomes reduced after treatment of 0.5 µg/ml C3 transferase or 0.5 µM H1152 for 8 h, respectively (*P<0.001, 2×2 χ^2^ test). (C) Concentration dependent suppression of multipolar mitosis in tetraploid p53^-/-^ MEF cells by C3 transferase, a cell permeable RhoA inhibitor. (D) Concentration dependent suppression of multipolar mitosis in tetraploid p53^-/-^ MEF cells by H1152, a cell permeable ROCK inhibitor. (C,D) Cells were induced to tetraploid cells with 0.5 µg/ml Cyto-B for 12 h, then were released and incubated with the indicated concentrations of the inhibitors, tracked by live cell imaging for 40 h (*P<0.001, 2×2 χ^2^ test). Error bars are standard deviation, from results of three independent experiments. n, the number of cells analyzed.

We next studied the mechanism by which p53 deficiency impairs the clustering of extra centrosomes and promotes multipolar mitosis in tetraploid cells. Recent studies have identified the critical roles of the actin cytoskeleton and its regulators, such as GTPases, in suppressing multipolar mitoses in cells with extra centrosomes [22]. In our study, the level of the activated form of RhoA, a GTPase, in p53^-/-^ MEFs was significantly higher than that in p53^+/+^ MEFs (Fig. 4A). Moreover, fluorescent Phalloidin-TRITC staining showed disorder of actin cytoskeleton in p53^-/-^ MEFs (Fig. S2), which could result from over-activated RhoA [31], [40]. It was reported that expression of epithelial cell transforming sequence 2 (ECT2), a guanine nucleotide exchange factor for RhoA, is repressed by p53 transcriptionally [41]. Consistent with this report, quantitative-PCR analysis showed that ECT2 mRNA levels is much higher in p53^-/-^ than p53^+/+^ MEFs (data not shown). The protein expression of ECT2 was also detected to be higher in p53^-/-^ than in p53^+/+^ MEFs, and knockdown of ECT2 in p53^-/-^ MEFs significantly reduced the activity of RhoA (Fig. 4A), corroborating that p53 could suppress RhoA activity via transcriptional regulation of ECT2 expression.

**Figure 5:**
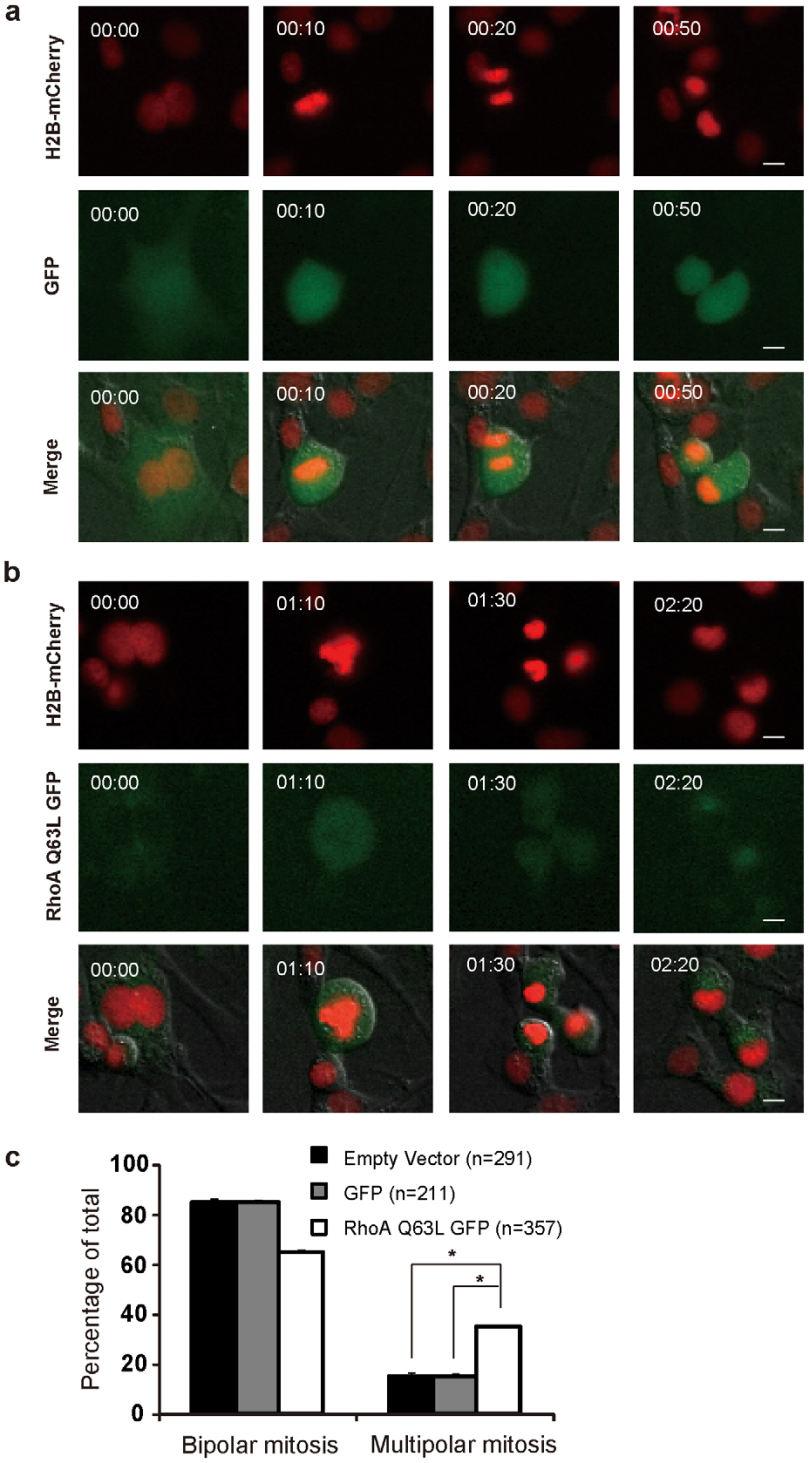
Increased RhoA activity increases multipolar mitosis in tetraploid cells with wild type p53. Mitotic polarity of cells was examined by live cell imaging after transient transfection of indicated plasmids followed by 1.5 µg/ml Cyto-B treatment for 12 h. (A,B) Selected serial images from time-lapse records are examples of: (A) Bipolar mitosis of a 3T3 cell expressing EGFP as a control; (B) Multipolar mitosis of a 3T3 cell expressing EGFP-RhoA-Q63L. In each image series, the upper panels (red) indicate chromosomal material, the middle panels (green) show whole cell distribution of EGFP or cytoplasmic distribution of EGFP-RhoA-Q63L respectively, and the bottom panels show images merged with red channel, green channel and differential interference contrast channel. Bar = 10 µm. Time counted from cell entering mitosis is presented as h:min at top-left. (C) The frequency of multipolar mitosis in tetraploid cells was increased after constitutively active RhoA expression in 3T3 cells (p53 normal) (*P<0.001, 2×2 χ^2^ test). Error bars are standard deviation, from results of three independent experiments. n, the number of cells analyzed.

We then assessed whether RhoA could influence the mitotic polarity of tetraploid cells. We studied the mitosis of tetraploid p53^-/-^ MEFs by live cell imaging after inhibition of RhoA activity using cell permeable C3 transferase. C3 transferase treatment suppressed multipolar mitoses in a concentration-dependent fashion in p53^-/-^ tetraploid MEFs (Fig. 4C). After treatment with 0.5 µg/ml of C3 transferase, multipolar mitoses decreased from 36±7% to 20±5% (Fig. 4C, p<0.001, 2×2 *χ^2^* test). However, when activity of RhoA was suppressed by 1 µg/ml C3 transferase, the percentage of multipolar mitosis increased again, indicating appropriate level of RhoA activity is essential for maintaining bipolar mitosis in tetraploid cells (Fig. 4C). Further higher concentration of C3 transferase (2 µg/ml) could even block the entry of mitosis. Moreover, we found a similar concentration-dependent V-type variation of multipolar mitosis in tetraploid p53^-/-^ MEFs after inhibiting the activity of ROCK, the downstream effector of RhoA, by the highly specific inhibitor H1152 (Fig. 4D). After treatment with 0.5 µM H1152, multipolar mitoses decreased from 38±5% to 17±5% (Fig. 4D, p<0.001, 2×2 *χ^2^* test). And, when activity of ROCK was suppressed by 1 µM H1152, the percentage of multipolar mitosis increased again. We then asked whether exogenously activated RhoA could promote multipolar mitosis in tetraploid cells. Induction of a constitutively active RhoA-GFP fusion protein (GFP-RhoA-Q63L, [42], [43]) in 3T3 cells significantly increased the proportion of multipolar mitosis (35±2% vs. 15±1% for cells expressing GFP only (p<0.001, 2×2 *χ^2^* test, Fig. 5) in tetraploid cells.

Next, we assessed whether overactivation of RhoA affected centrosome clustering or inactivation. We studied centrosome behaviors at metaphase of p53^-/-^ tetraploid MEFs after inhibition of RhoA activity using C3 transferase or inhibiting ROCK by H1152. After treatment with 0.5 µg/ml C3 transferase or 0.5 µM H1152, multipolar spindles decreased from 48±3% to 23±3% or 26±5% (Fig. 4B, p<0.001, 2×2 *χ^2^* test) respectively, which was consistent with previous results from live cell imaging. Further investigation showed that 73.1±2.3% of p53^-/-^ tetraploid MEFs displayed centrosome clustering while the proportion increased significantly after C3 transferase or H1152 treatment (86.6±5.3%, 81.0±3.5%, respectively) (Table 3, p<0.05, 2×2 *χ^2^* test). However, the proportion of cells with inactivated centrosomes was similar, 21.5±2.4% in control cells and 19.7±3.5% after C3 transferase treatment or 25.4±0.9% after H1152 treatment. The treated cells had a much lower proportion of cells without clustered or inactivated centrosomes than untreated counterparts (17.4±2.4% in control, 8.5±3.5%, 10.4±1.4% after C3 transferase or H1152 treatment, respectively, Table 3, p<0.05, 2×2 *χ^2^* test). C values and Cl values in treated cells were significantly higher than those untreated control cells (p<0.001, t-test, Table 4), indicating that the ability to cluster extra centrosomes was impaired when p53 was lost. In contrast, the I value was similar between treated and untreated cells.

**Table 3:** Centrosome clustering was enhanced in p53^-/-^ MEF cells with extra centrosomes after RhoA or ROCK inhibition.

	No. of cells analyzed	% (Mean±s.d.)			
		Centrosome inactivation	Centrosome clustering	Both	Neither
Control	316	21.5±2.4	73.1±2.3	12.0±2.3	17.4±2.4
C3 transferase	284	19.7±3.5	86.6±5.3 *	14.8±0.7	8.5±3.5 *
H1152	347	25.4±0.9	81.0±3.5 *	16.7±1.5	10.4±1.4 *

p53^-/-^ MEF cells were treated with Cyto-B (0.5 µg/ml) for 12 h, then released and treated with 0.5 µg/ml C3 transferase (RhoA inhibitor) or 0.5 µM H1152 (ROCK inhibitor) for 8h, and stained with antibodies against γ-tubulin and α-tubulin for centrosomes and microtubules, respectively. Nuclei were stained with DAPI. Note that only cells with more than 3 centrosomes at metaphase were analyzed. The percentage of cells with indicated mechanism was shown separately. Mean ± s.d. from three independent experiments.

*p<0.05, 2×2 χ^2^ test, compared with control cells.

**Table 4:** Centrosome behavior after RhoA or ROCK inhibition in p53^-/-^ MEF cells with extra centrosomes.

	No. of cells analyzed	Mean±s.d.		
		C value	Cl value	I value
Control	316	1.69±0.56	1.58±0.53	0.06±0.12
C3 transferase	284	1.93±0.59 *	1.81±0.58 *	0.06±0.12
H1152	347	1.90±0.55 *	1.76±0.56 *	0.07±0.13

*p<0.001, t-test, compared with the control.

**Figure 6:**
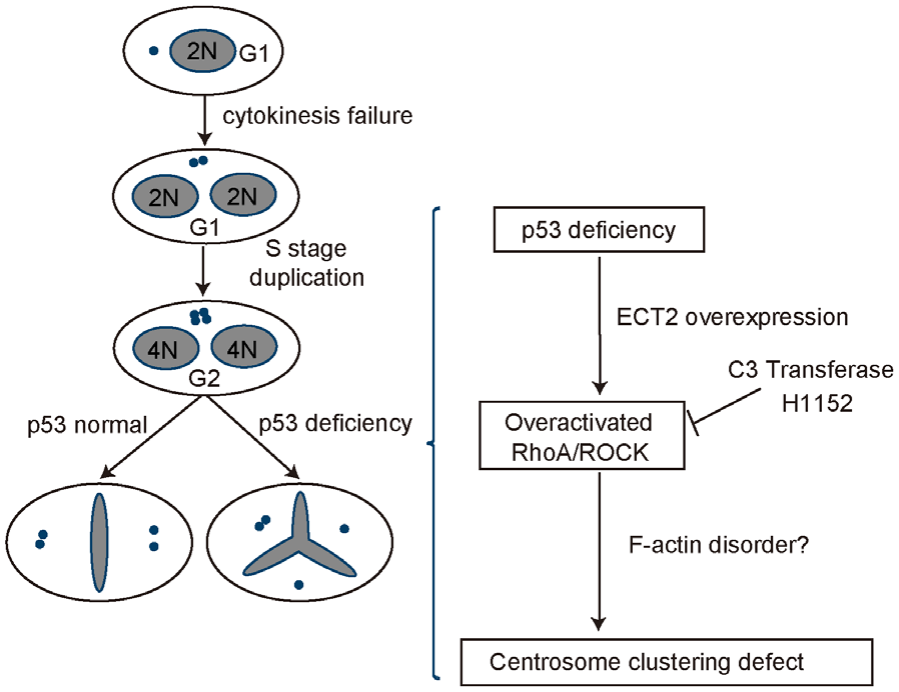
p53 deficiency contributes to multipolar mitosis in tetraploid cells. The model shows that p53 abnormality is a major cause for multipolar mitosis in tetraploid cells. Specifically, we propose that a number of distinct primary cell cycle errors such as cytokinesis failure leads to tetraploid cells with 4N DNA and two centrosomes. These cells undergo DNA replication and centrosome duplication before entering mitosis, giving rise to octoploidy with four centrosomes. In p53 proficient cells, most of them will undergo bipolar mitosis, most likely through clustering extra centrosomes, though a mechanism inactivating extra centrosomes may also occur. In the absence of a functional p53, however, ECT2 is overexpressed, and then RhoA/ROCK is overactivated, which impairs the ability of clustering extra centrosomes most likely through disrupting the arrangement of actin cytoskeleton and leads to multipolar mitosis, giving rise to chromosomal instability and aneuploidy.

Taken together, our results demonstrate that overactivation of RhoA accounts for impairment of ability to cluster extra centrosomes and consequently increase multipolar mitosis in p53 abnormal tetraploid cells (Fig. 6).

## Discussion

Aneuploidy is a key feature of most human cancer, and is proposed to result from a metastable state of tetraploidy [2], [3]. Over half of human cancers have loss or mutation of the p53 gene [44], [45], and it has been estimated that 80% of human tumors have defects in p53 signaling pathways [46]. There was a significant association between p53 abnormalities and genetic instability in human tumors [47], [48]. So, whether and how p53 influences the transformation of tetraploidy to aneuploidy is very attractive.

Given that inactivation of p53 in tetraploid cells results in progression toward aneuploidy and tumorigenesis [9], a p53 dependent “tetraploidy checkpoint” which could prevent the tetraploid cells from entering mitosis has been proposed [4], [49]. On the other hand, FACS analysis showed that there are much more apoptotic cells in nocodazole induced p53 proficient tetraploid cells than in their p53 deficient counterparts [12], [13]. However, by live cell imaging, we showed that most tetraploid cells (>86%) could enter mitosis regardless of the status of p53, this is in accordance with previous reports [16], [17]. What's more, we showed that the percentage of p53 proficient and deficient tetraploid cells entering mitosis was not significantly different, and there was not even a p53-dependent arrest of cell cycle (Fig. 1A,B,C,D). On the other hand, the percentage of cells undergoing cell death in both p53^+/+^ and p53^-/-^ tetraploid MEF cells were low (3.7% for p53^+/+^ and 1.8% for p53^-/-^) and not significantly different (p>0.05, 2×2 *χ^2^* test, Fig. 1C). We never observed cell death in tetraploid N/TERT-1 and N/TERT-1 p53DD cells during live cell imaging (Fig. 1B) and others report no cell death in p53 normal tetraploid hepatocytes during live cell imaging of the cells [50]. Thus, p53 per se may not impede cell cycle or induce cell death. The previously reported “tetraploidy checkpoint” and p53 dependent cell death in tetraploid cells may be either an artifact of the relatively high concentration of nocodazole used to induce tetraploidy or could be explained by cell-type-specific differences. Alternatively, these apoptotic cells detected by FACS analysis could be the daughter cells from multipolar mitosis of p53 proficient tetraploid cells, as these daughter cells inherited extremely disordered genome content, which would lead to apoptosis especially in p53 proficient cells.

To induce multipolar mitosis, cells should have extra centrosomes and deficiency of clustering/inactivating extra centrosomes [21]. As tetraploid cells often result from cytokinesis failure, endoreduplication or cell fusion, they own doubled number of centrosomes. So, the factors that influence centrosome clustering and inactivation are critical for preventing multipolar mitosis in tetraploid cells. Our research indicated that p53 deficiency impaired centrosome clustering but not inactivation of extra centrosomes in tetraploid cells (Table 1,2 and supplemental Table S1, S2), and then promoted multipolar mitosis. Centrosome clustering depends on centrosome movement [22]. Some F-actin cables are concentrated around the centrosomes and extend between the centrosomes and the cell cortex [51]. Normal F-actin is required for centrosome movement [51], [52], [53], [54], [55]. Myosin II is also required for centrosome movement and positioning [56]. Thus, normal F-actin organization and Myosin II is important for normal centrosome clustering. As RhoA/ROCK is known to regulate actin cytoskeleton and myosin light-chain phosphorylation [57], [58], [59], overactivated RhoA in tetraploid cells with p53 abnormalities would disrupt centrosome clustering. However, disruption of F-actin does not impair the spindle pole integrity and spindle length [51], indicating that the ability of centrosome to assemble and organize microtubules was not affected, implying that overactivated RhoA induced disorganized F-actin would not influence the inactivation of extra centrosomes. Other factors could also influence multipolar mitosis. Overactivated p38α, which usually is thought to phosphorylate and activate p53, in p53-/- tetraploid cells is involved in multipolar mitosis [60]. Actually, RhoA could activates p38 [61], and these results are in accordance with our finding that overactivated RhoA promoted multipolar mitosis. In a recent research [18], after long time culturing, the descendant cells from p53^-/-^ HCT116 tetraploidy have a higher proportion of cells with centrosome amplification than that from p53^+/+^ HCT116 tetraploidy, which induced more multipolar mitosis in p53^-/-^ cells. This indicated that p53 may also influence the survivability of cells with extra centrosomes. Actually, they found that there were overexpressed Mos, which could promote multipolar mitosis, in some p53^-/-^ clones form tetraploid cells. As overexpressed Mos could induce cell death in p53^+/+^ cells, all p53^+/+^ cell clones and some p53^-/-^ cell clones which did not overexpress Mos could not induce high incidence of multipolar mitosis.

We showed that p53 deficiency in MEFs led to a significant up-regulation of RhoA activity (Fig. 4A) as reported previously [31], [32]. Furthermore, the knockout of p53 in HCT116 induced similar elevation of activated RhoA, and p53 mutations in HCT116 and U2OS also overactivated RhoA [33], [34], [35]. Though it is very consensus in the literature that p53 deficiency leads to increased levels of active RhoA, the molecular details of how this occurs varies between studies. Both increased activity of PI 3-kinase and upregulation of ECT2, a guanine nucleotide exchange factor for RhoA, have been proposed to be responsible for increased levels of active RhoA in p53 deficient cells [32], [41]. Additionally, the atypical Rho protein RhoE (or Rnd3) is a p53 target gene that opposes activation of RhoA [62], [63]. Our experiments showed that ECT2 was overexpressed in p53^-/-^ MEFs compared with p53^+/+^ MEFs, and knockdown of ECT2 by RNAi reduced the ability of ECT2 to overactivate RhoA in p53^-/-^ MEFs (Fig. 4A). This indicates that high level of ECT2 is responsible for the abnormal high activity of RhoA in p53^-/-^ MEFs. High levels of RhoA could disrupt the assembly of actin cytoskeleton [40]. It is likely that over-activated RhoA could also do the same (Fig. S2) [31]. Knockdown of proteins involved in the organization and regulation of the actin cytoskeleton or disruption of F-actin itself could promote multipolar mitosis in cells with extra centrosomes [22], [64]. Thus, our data imply a “p53/RhoA/F-actin” pathway which works to regulate multipolar mitosis in cells with extra centrosomes. Interestingly, overinhibition of RhoA activity also promotes multipolar mitosis (Fig. 4C,D), in line with the report that knockdown of rho, the homolog of RhoA in drosophila, induced multipolar mitosis in S2 tetraploid cells [22]. This indicates that appropriate activity of RhoA is required for preventing tetraploid cells from multipolar mitosis.

The fate of tetraploid cells is important, given that the human body contains millions of dividing cells at any time and many abnormalities could disrupt the mitosis and lead to tetraploid cells. Firstly, deregulation of many proteins such as deficiency of myosin light-chain phosphorylation, overexpression of Aurora A, and mutation/inactivation of many tumor suppressers such as APC, BRCA1, and LATS2 could induce tetraploid cells [65], [66], [67], [68], [69]. Secondly, some virus, bacteria, entosis and nondisjunction in mitosis could also induce tetraploid cells [37], [70], [71], [72]. Thirdly, DNA damage in mitosis could also induce tetraploid cells. Activation of the ATM and ATR dependent DNA damage response impairs spindle assembly in mitosis, leading to the formation of disorganized microtubule and eventually tetraploid cells if the DNA damage were not repaired in time [73], [74]. As an intermediate, tetraploid cells could generate aneuploid cells through either progressive chromosomal loss [75] or multipolar mitoses in which chromosomes are randomly distributed among more than two daughter cells [18], [76]. In this study, we showed that p53 abnormality could significantly promote multipolar mitosis, leading to aneuploid daughter cells. Actually, a recent study showed that high Aurora-A expression, which could induce tetraploidy [66], alone is not associated with overt multipolar mitoses, additional p53 mutations are necessary for this to occur, at least in invasive esophageal cancer cells [77]. Although most daughter cells from multipolar mitosis would die during the subsequent interphase or after one additional round of mitosis [75], a small percentage of aneuploid daughter cells inheriting a suitable genome composition could survive and even compete with normal cells, as observed in cell cultures and in tumors [18], [76]. And this would be more realistic in p53 deficient tetraploid cells, in which doubling of the genome increases the probability that some daughter cells of multipolar division will have enough chromosomes to remain viable and p53 deficiency leads to apoptosis resistance. Other than apoptosis, multipolar mitosis induced aneuploid cells maybe also activate a p53-p21 driven premature senescence phenotype. Actually, these proteins playing role in centrosome and kinetochore integrity and in particular of centrosomal TACC (transforming acidic coiled coil) proteins are linked to senescence via p53 [78]. Meanwhile, these proteins also regulate centrosome clustering and multipolar mitosis [22], [79].

Taken together, here we show that p53 could not significantly affect mitotic entry and cell death of tetraploid cells, while p53 abnormality promotes multipolar mitosis by impairing centrosome clustering via modulation of the RhoA/ROCK signaling pathway in tetraploid cells, leading to aneuploidy. Loss of p53 function is favorable for the survival of aneuploid cells [80], [81] and our results described here demonstrate that loss of p53 function additionally promotes their formation by inhibiting centrosome clustering. This provides an alternative explanation for arising of aneuploidy during tumour initiation and the frequent coexistence of p53 abnormalities, genomic instability, and aneuploidy in tumors.

## Materials and Methods

### Generation of p53^+/+^ and p53^−/−^ MEF

The collection and using mouse tissue were under the approval of the Institutional Review Board at University of Science and Technology of China, the approval ID: USTCAU201000004. C57B1/6J mice heterozygotic for p53 were purchased from The Jackson Laboratory. Mice were mated and at 13-days gestation, the female was killed. Embryos were washed in PBS, the heads and internal organs were isolated for p53 genotype analysis, and the remainder was grinded to obtain dispersed cells. Cells were placed on a 10-cm plate coated with 0.1% gelatin and grown in DMEM (Gibco Life Technologies, Grand Island, NY, USA) containing pen-strep (Gibco Life Technologies, Grand Island, NY, USA) and 10% FBS (Hyclone Labs Inc., Logan, Utah, USA) in a humid incubator at 37°C in 5% CO_2_, as described [82]. Only passage 1 or 2 cells were used for further experiments. Cells were genotyped by PCR according to instructions from The Jackson Laboratory.

### Cell Lines

N/TERT-1 and N/TERT-1 p53DD cells were kind gifts from James Rheinwald (Department of Dermatology, Harvard Medical School, USA) and cultured as described [36]. HeLa cells (CCL2 from ATCC), H1299, HCT116, NIH 3T3 were cultured in DMEM (Gibco Life Technologies, Grand Island, NY, USA) supplemented with 10% fetal bovine serum (Hyclone Labs Inc., Logan, Utah, USA) and pen-strep (Gibco Life Technologies, Grand Island, NY, USA). N/TERT-1 and N/TERT-1 p53DD cells expressing GFP-H2B and HeLa, H1299, HCT116, 3T3 cells expressing mCherry-H2B were created as described [37]. All cells were maintained in a humid incubator at 37°C in 5% CO_2_.

### Live cell imaging

Cancer cell lines (HeLa, H1299, and HCT116) and N/TERT-1 series cells were grown on 4-well coverglass bottom chamber (Lab-Tek II, Nalge Nunc International, Roskilde Denmark) for 24 h or 3 d. Then cells are treated with cytochalasin B (Sigma, St. Louis, MO, USA) for 24 h (cancer cell lines, 3 µg/ml; N/TERT-1 series, 6 µg/ml). To terminate drug treatments, cells were washed with drug-free medium more than five times over a period of 30 min before imaging. Images were acquired automatically at multiple locations on the coverslips using a Nikon TE2000E inverted microscope fitted with a 20× Nikon Plan Fluor objective (Nikon Corporation, Tokyo, Japan), a linearly-encoded stage (Prior Scientific Instruments Ltd., Cambridge, UK) and a Hamamatsu Orca-ER CCD camera (Hamamatsu Photonics, Hamamatsu, Japan). Fluorescence illumination used a mercury-arc lamp with two neutral density filters (for a total 32-fold reduction in intensity). The microscope was controlled using NIS-Elements Advanced Research (Nikon Corporation, Tokyo, Japan). The microscope was housed in a custom-designed 37°C chamber with a secondary internal chamber that delivered humidified 5% CO_2_. Fluorescence and differential interference contrast images were obtained every 10∼15 min for a period of 50–64 h. For MEF series, cells were seeded on 8-well coverglass bottom chamber, coated with 0.1% gelatin, for 24 h (2×10^4^ cells per well). After 0.5 µg/ml cytochalasin B treatment for 12 h, cells were washed with drug-free medium more than five times over a period of 30 min to terminate drug treatments before imaging. Then images were acquired as described above and differential interference contrast images were obtained every 10 min for a period of 40 h. For experiments in Fig. 4, cell permeable RhoA inhibitor C3 transferase (Cytoskeleton, Denver, CO, USA) or ROCK inhibitor H1152 (Calbiochem, La Jolla, CA, USA) was added to the culture medium before live cell imaging. C3 transferase was dissolved in 50% glycerol (Invitrogen, Carlsbad, CA, USA). Standard deviations were derived from the results from at least 3 independent experiments.

### Plasmid and DNA transfections

pcDNA3-EGFP and pcDNA3-EGFP RhoA-Q63L were kind gifts from Professor Gary Bokoch (Departments of Immunology & Microbial Science, The Scripps Research Institute, USA) [83]. pEGFP-p53 R175H was kind gift from Professor Mian Wu (School of Life Sciences, University of Science and Technology of China) [84]. 3T3 H2B-mCherry was seeded on 4-well coverglass bottom chamber (1.5×10^4^ cells in each well) and cultured with DMEM (10% FBS). After 24 hours, cells were transfected with plasmid (1 µg for each well) using the Lipofectamine 2000 transfection reagent (Invitrogen, Carlsbad, CA, USA) (2 µl for each well) following the manufacture's protocol. Fresh medium was added 4 hours later. Cells were cultured for 36 hours after transfection then treated with 1.5 µg/ml cytochalasin B (Sigma, St. Louis, MO, USA) for 12 h. To terminate drug treatments, cells were washed with drug-free medium more than five times over a period of 30 min before imaging. Live cell imaging were performed as described.

### RNA Interference

p53^-/-^ MEF cells were cultured as described previously [82]. ECT2 siRNA corresponding to nucleotide sequences 867–891 (GenBank accession no. BC045614) and the control siRNA were chemically synthesized by Genepharma (Shanghai, China). Cells were transfected with Lipofectamine RNAiMAX Reagent (Invitrogen, CA, USA) and 20 nM siRNA according to the manufacturer's protocol and cultured in growth medium for up to 48 h. The efficiency of the knockdown was examined by western blotting analysis using rabbit anti-Ect2 antibody (Santa Cruz, Santa Cruz, California, USA).

### RhoA activity assay

The GST-Rhotekin which contains the RhoA interactive domain was expressed and purified in Escherichia coli, which is a kind gift from Professor Aihua Gong (School of Medical Science and Laboratory Medicine, Jiangsu University, China). The Rho GTPase activities were processed as previously described [85]. Briefly, wild-type and p53^-/-^ MEF cells grown in log phase were used. After the indicated treatment, cells were lysed before incubation with GST-Rhotekin, coupled to glutathione-agarose beads (GE Healthcare, Piscataway, NJ, USA). After precipitation, complexes were washed four times with wash buffer, eluted in SDS–PAGE sample buffer, immunoblotted and analysed with antibodies against RhoA (Santa Cruz, Santa Cruz, California, USA). Aliquots taken from supernatants prior to precipitation were used to quantify total RhoA present in cell lysates. All experiments were repeated three times, and the bands were quantified using the Gel-Pro Analyzer 4.0 computer program (Media Cybernetics, Silver Spring, MD).

### Immunofluorescence

MEF cells at passage 1 were grown on glass coverslips coated with 0.1% gelatin for 24 h, treated with 0.5 µg/ml cytochalasin B for 12 h to induce tetraploid cells, then released or treated with 0.5 µg/ml C3 transferase or 0.5 µM H1152 for 8 h after washing with drug-free medium more than five times over a period of 30 min to terminate drug treatments. Then, cells were permeabilized with 0.5% Triton X-100 (Sigma, St. Louis, MO, USA) in Perm buffer (100 mM PIPES (Sigma, St. Louis, MO, USA), 10 mM EGTA (BioBasic, ED0077), 1 mM MgCl_2_ (Sigma, St. Louis, MO, USA)), and fixed in methanol for 30 min at −20°C. The coverslips were hydrated in TBS/0.1% Triton X-100 (TBST) for 3×5 min and blocked with 2% BSA in TBST for 1 h, then subjected to indirect immunofluorescence with the following primary antibodies: rabbit Abs to gamma-tubulin (Sigma, St. Louis, MO, USA) at 1∶200, mouse Abs to alpha-tubulin (Sigma, St. Louis, MO, USA) at 1∶200. The coverslips with primary antibodies was placed in a humid chamber at 37°C for 3 h and then washed with TBST for 3×10 min. The secondary antibodies were Alexa Fluor 488-conjugated donkey anti-mouse IgG (1∶200, Molecular Probes A21202), Alexa Fluor 555 -conjugated donkey anti-rabbit IgG (1∶100, Molecular Probes A31572) at 37°C for 2 h. In all cases, the coverslips were counterstained with DAPI at 3 µg/ml. Samples were scored using a BX-61 microscope (Olympus, Osaka, Japan). At least 100 cells were scored per experiment, and each experiment was repeated three times.

### Statistical analysis

Unless otherwise specified, all experiments were carried out in triplicate parallel instances and independently repeated at least three times. Data were analyzed with SPSS version 10 (SPSS, Chicago, Illinois, USA) and statistical significance was assessed by means of two-tailed Student's t-test or 2×2 *χ^2^* test. Values of p<0.05 were considered statistically significant.

## Supporting Information

Figure S1 **Etoposide increases the level of p53 and its downstream proteins in NIH3T3 cells. **Cells were seeded and cultured in 6-well plates until at ∼60% confluency, then treated with 20 µM etoposide, an inhibitor of topoisomerase II that induces DNA damage, for 12h. DMSO was used as a control. Then western blotting was carried out to detect the expression of p53 and its direct downstream protein p21and Bax, which are related to cell cycle arrest and apoptosis respectively. β-actin was used as a loading control.
(TIF)

Figure S2 **Loss of p53 disrupts actin cytoskeleton.** Cells were plated on 0.1% gelatin-coated coverslips and cultured for 48 h and then stained with Phalloidin-TRITC to visualize the F-actin cytoskeleton (Red). Nuclei were counterstained with DAPI (blue).
(TIF)

Table S1 Centrosome clustering was impaired in p53-/- MEF cells with four centrosomes.
(PDF)

Table S2 Centrosome behavior of MEF cells with four centrosome.
(PDF)
