# Supplementary figures and images for: p53 Dependent Centrosome Clustering Prevents Multipolar Mitosis in Tetraploid Cells
Source: PLoS One. 2011 Nov 4;6(11):e27304. doi: 10.1371/journal.pone.0027304 (PMC3208627; doi:10.1371/journal.pone.0027304)

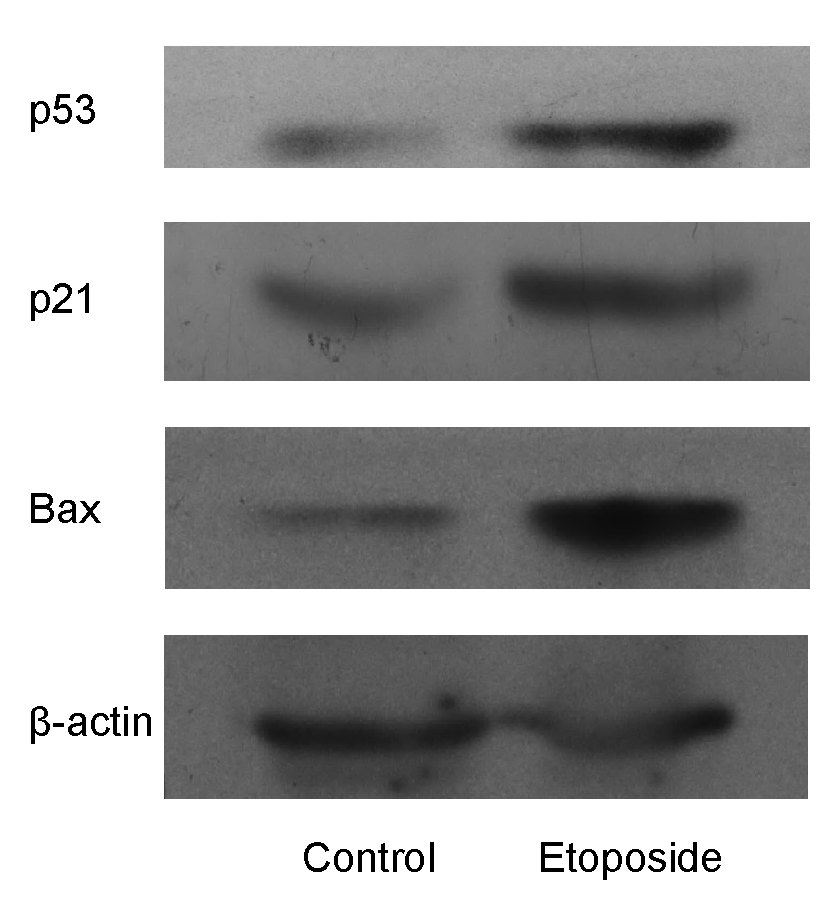

Supplement: Figure S1 — Etoposide increases the level of p53 and its downstream proteins in NIH3T3 cells. Cells were seeded and cultured in 6-well plates until at ∼60% confluency, then treated with 20 µM etoposide, an inhibitor of topoisomerase II that induces DNA damage, for 12h. DMSO was used as a control. Then western blotting was carried out to detect the expression of p53 and its direct downstream protein p21and Bax, which are related to cell cycle arrest and apoptosis respectively. β-actin was used as a loading control. (TIF) [file pone.0027304.s001.tif]

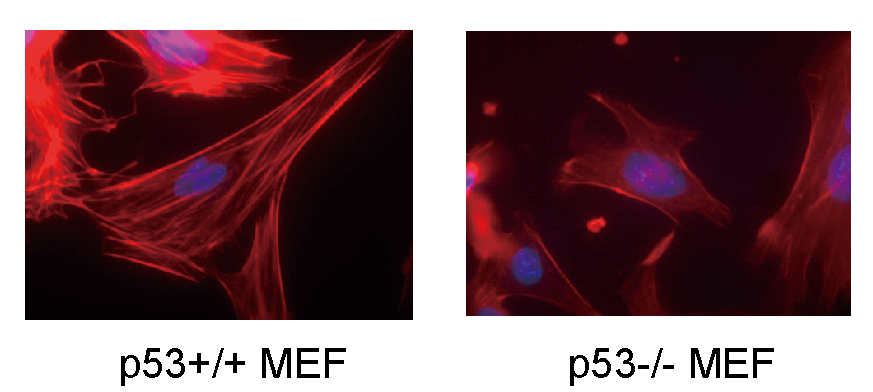

Supplement: Figure S2 — Loss of p53 disrupts actin cytoskeleton. Cells were plated on 0.1% gelatin-coated coverslips and cultured for 48 h and then stained with Phalloidin-TRITC to visualize the F-actin cytoskeleton (Red). Nuclei were counterstained with DAPI (blue). (TIF) [file pone.0027304.s002.tif]
