# Supplementary material for: p53 Dependent Centrosome Clustering Prevents Multipolar Mitosis in Tetraploid Cells
Source: PLoS One. 2011 Nov 4;6(11):e27304. doi: 10.1371/journal.pone.0027304 (PMC3208627; doi:10.1371/journal.pone.0027304)
Supplement: Table S1 — Centrosome clustering was impaired in p53-/- MEF cells with four centrosomes. (PDF) [file pone.0027304.s003.pdf]

Supplementary table 1. Centrosome clustering was impaired in p53$^{-/-}$ MEF cells with four centrosomes

	No. of cells analyzed	% (Mean±SD)			
		Centrosome inactivation	Centrosome clustering	Both	Neither
p53$^{+/+}$ MEF	177	23.2±9.2	93.8±4.0	20.3±7.8	3.4±1.8
p53$^{-/-}$ MEF	308	25.3+3.0	76.3+8.5 *	13.6+10.1	11.7+9.3 *

Cells were treated with Cyto-B (0.5 μg/ml) for 12h, then released for 8h, and stained with antibodies against γ-tubulin and α-tubulin for centrosomes and microtubules, respectively. Nuclei were stained with DAPI. Note that only cells with four centrosomes at metaphase were analyzed. The percentage of cells with indicated mechanism was shown separately. Mean ± SD from three independent experiments.

* $p<0.001$, 2×2 χ^2 test, compared with MEF p53$^{+/+}$ cells.
