# Supplementary material for: p53 Dependent Centrosome Clustering Prevents Multipolar Mitosis in Tetraploid Cells
Source: PLoS One. 2011 Nov 4;6(11):e27304. doi: 10.1371/journal.pone.0027304 (PMC3208627; doi:10.1371/journal.pone.0027304)
Supplement: Table S2 — Centrosome behavior of MEF cells with four centrosome. (PDF) [file pone.0027304.s004.pdf]

Supplementary table 2. Centrosome behavior of MEF cells with four centrosomes

	No. of cells analyzed	Mean±SD		
		**C value** [a]	**CI value** [b]	**I value** [c]
$p53^{+/+}$ MEF	177	1.91±0.25	1.78±0.32	0.07±0.14
$p53^{-/-}$ MEF	308	1.66±0.39 *	1.53±0.40 *	0.07±0.14

* p<0.001, t-test, compared with MEF $p53^{+/+}$ cells.

a C value (the number of centrosomes / the number of spindle poles) indicates the ability of maintaining bipolar spindle in cells with extra centrosomes;

b Cl value (the number of activated centrosomes / the number of spindle poles) indicates the ability of clustering centrosomes in cells with extra centrosomes;

c I value (the number of inactivated centrosomes / the number of total centrosomes) indicates the ability of inactivating extra centrosomes in cells with extra centrosomes.
